# Extracellular matrix stiffness aggravates urethral stricture through Igfbp3/Smad pathway

**DOI:** 10.1038/s41598-023-41584-6

**Published:** 2023-08-31

**Authors:** Kaixuan Li, Ke Ding, Quan Zhu, Feng Han, Xi He, Shuo Tan, Ziqiang Wu, Zhihuan Zheng, Zhengyan Tang, Yanling Liu

**Affiliations:** 1 Department of Cardiac Surgery, Xiangya Hospital, Central South University, Changsha, 410008 Hunan China; 2 Present Address: Department of Urology, Xiangya Hospital, Central South University, 87 Xiangya Road, Changsha, 410008 Hunan China; 3 Present Address: Provincial Laboratory for Diagnosis and Treatment of Genitourinary System Disease, 87 Xiangya Road, Changsha, 410008 Hunan China; 4 Department of Orthopedics, The First Affiliated Hospital, Medical College of Zhejiang University, Hangzhou, 310003 Zhejiang China; 5 Department of Urology, Third Xiangya Hospital, Central South University, Changsha, 410013 Hunan China

**Keywords:** Urology, Urethra

## Abstract

Urethral stricture refers to the narrowing of the urethral lumen. While previous studies have hinted at inflammation as the initial driver of this condition, the reasons and mechanisms behind its progression remain largely unknown. By Atomic force microscope (AFM), researchers measured the matrix stiffness of urethra to be 5.23 ± 0.37 kPa for normal tissue and 41.59 ± 2.48 kPa for stricture urethral scar. Similar results were observed in rat urethral stricture models, where the matrix stiffness of normal urethra was 4.29 ± 0.82 kPa, while 32.94 ± 7.12 kPa for urethral stricture scar. Notably, the matrix stiffness increased in rat models over time. To further investigate, polyacrylamide hydrogels were employed to mimic different levels of stiffness for normal and stricture condition. Interestingly, higher matrix stiffness led to an increased fibroblast-to-myofibroblast transition (FMT) in rat urethral fibroblasts, indicated by enhanced expression of α-SMA and Collagen I, as well as changing in the morphology of fibroblast. RNA-seq analysis suggested that Igfbp3/Smads might regulate the progressive FMT in urethral stricture. In the experiment where the expression of Igfbp3 was inhibited, increasing matrix stiffness lose the potential to stimulate FMT progression and the expression of p-Smad2/3 decreased. On the contrary, overexpression of Igfbp3 promoted the process of FMT in urethral fibroblasts. In conclusion, Igfbp3/Smad pathway appeared to be involved in the progression of urethral fibrosis. This finding suggested that Igfbp3/Smad might be an promising target for future research and treatment in this filed.

## Introduction

Urethral stricture refers to the narrowing of the urethral lumen, accompanied by the presence of fibrous scar lesions in the mucosa and surrounding cavernous tissue. Urethral stricture is a common disease, with an incidence rate ranging from 0.2 to 1.2%^1^. As urethral stricture advances, it can give rise to bladder decompensation and vesicoureteral reflux, and even more severe complications such as renal dysfunction and hydronephrosis, which pose significant threats to the well-being of affected patients^2–4^. Despite the most appropriate treatments, the recurrence and progression of urethral stricture present daunting challenges for urologists^5^. Similar to the management of various solid organ fibrosis, such as pulmonary fibrosis and renal fibrosis, there is currently no pharmaceutical intervention capable of completely reverse urethral fibrosis. Therefore, studying the progressive pathogenesis of urethral stricture and exploring novel therapeutic targets and medications hold promise as a novel avenue for preventing urethral stricture, delaying recurrence, and improving the quality of life of affected patients.

Fibroblast-to-myofibroblast transition (FMT) plays a pivotal role in the pathogenesis of fibrosis, encompassing various conditions such as myocardial fibrosis, lung fibrosis, renal fibrosis etc^6–9^. The heightened secretion of extracellular matrix (ECM) by myofibroblasts leads to escalating tissue stiffness, thereby contributing to the progression of fibrosis^6, 10, 11^. Research has indicated that under physiological conditions, the stiffness of lung and liver tissues ranges from 0.5 to 1 kPa. However, in tissues affected by pulmonary fibrosis, where an elevated FMT level has been observed, the matrix stiffness exhibits a significant increase, reaching values between 25 and 100 kPa, in a time-dependent manner^12, 13^. Similar outcomes have been observed in hepatic fibrosis^14^, myocardial fibrosis^15^, renal fibrosis^16^, ect. Although several studies have demonstrated the association between fibrosis progression and increasing stiffness, the mechanism remained largely unknown, especially in urethral fibrosis^17–23^. Therefore, further investigation is warranted to elucidate the intricate processes governing this phenomenon in urethral fibrosis and enhance our understanding of its pathogenesis.

In this study, we measured the precise stiffness of normal and stricture urethra in both human and rat models. Further, we investigated the potential of increased matrix stiffness, occurring subsequent to initial fibrosis, to exacerbate the fibrotic process via the Igfbp3 pathway. Consequently, the primary focus of this investigation is to examine the impact of matrix stiffness on the advancement of urethral stricture, thus establishing a fundamental groundwork for the subsequent identification of potential drug targets for this condition.

## Materials and methods

### Immunohistochemistry and immunofluorescence

The normal urethral tissues of the human were from penile cancer (without invading the tunica albuginea and urethral tissues, with lesions confined to the glans and Foreskin), the patient underwent partial penectomy, and the urethral tissue was used as the control group; the stricture urethral tissue was obtained from urethral stricture patients who suffered from external urethral trauma during the periods from June 30, 2021 to June 30, 2022. Only males aged between 18 and 65 were selected for this study. These patients were diagnosed with posterior urethral stricture based on clear diagnostic criteria. The underlying cause of this condition was found to be pelvic fractures resulting from car accidents. This study was approved by the Medical Ethics Committee of Xiangya Hospital, Central South University (202112612). After deparaffinization and hydration, 3% H_2_O_2_ solution was used to remove endogenous catalase, sodium citrate was used for antigen retrieval, goat serum was used for blocking, primary antibody treated overnight at 4 °C. After adding the secondary antibody, the DAB color was developed.

### Atomic force microscope (AFM) measurement of matrix stiffness

The rats were anesthetized with 1% sodium pentobarbital solution. The urethral tissue or urethral stricture tissue of the rats were extracted and placed on the AFM microscope. On the holder, calibrate the probe in the air to obtain the spring constant of the probe: 92.04 N/nm, place the calibrated probe in a water environment, and perform the optical lever sensitivity calibration of the probe again, Amp InvOLS: 38.56 nm/V. In contact mode, force curve measurement, force curve speed: 1 Hz. In Asylum software, Young's modulus was automatically fitted to the needle insertion curve of 256 force curves within the mapping range in Hertz model, in which the ball material: polystyrene ball with a diameter of 12 µm; the elastic coefficient of the probe: 0.05 N/m; the probe frequency in the air: 22 kHz.

### Animal and urethral stricture modeling

The animal experiments were approved by the Animal Ethics Committee of Central South University, and conformed to the relevant regulations on animal ethics such as animal protection, animal welfare and ethical standards (No: 2021921). The rats were provided by Changsha Tianqin Biotechnology Co., Ltd., and were 10-week-old male Sprague Dawley (SD) rats weighing 350–450 g. Rats were cultured in a specific pathogen free (SPF) barrier with constant temperature and humidity (22–24 °C, 70% humidity), and all rats could eat and drink freely.

Twelve male SD rats were randomly divided into 2 groups, namely the control group and the urethral stricture group, with 6 rats in each group. Rats were marked respectively, and anesthetized with 1% sodium pentobarbital (50 mg/kg). The epidural catheter was inserted into the rat urethra which help to visualize the urethra when the ventral penile skin was removed. Rats underwent 4 partial incisions of the penile urethra with a 23G needle. The rats in the urethral stricture group were injected with TGFβ1 (1 µg) dissolved in 100 µL of saline around incisions, however, the rats in the control group were injected with saline and underwent sham operation. In order to reduce the mortality of the rats, a cystostomy (suture the catheter at the bladder, and place the catheter subcutaneously into the skin stoma behind the neck of the rat) was performed in each rat. The epidural catheter was then removed and the penile skin was closed with 5–0 absorbable suture. Rats were fed for 1 month^24^.

Furthermore, all animal experiments were performed in accordance with relevant guidelines and regulations and reporting in the manuscript follows the recommendations in the ARRIVE guidelines 2.0.

### Ultrasound and cysto-urethrography

The rats were anesthetized with 1% sodium pentobarbital (50 mg/kg), and the loach guide wire was inserted from the external urethra of the rat to determine the position of the urethra. As for cysto-urethrography, the Iohexol contrast agent was injected through the ostomy tube. All above were finished by professional radiologists.

### Isolation of primary rat urethral fibroblasts

For fibroblasts isolation, we usually use 60–80 g rats. Simply put, after separation, cutting, digestion, and filtration, we collected about 10^4^ cells from one rat and all those cells were plated in one well of a 6-well plate. 8 h after first plating, un-attached cells (mostly endothelial cell) were removed by replacing with new medium. And these cells could be passaged 3 times (in 1:3 ratio).

### Preparation of polyacrylamide hydrogels with different matrix stiffness

Immerse the cell slides in a 6 cm dish containing 0.1 mol/L NaOH, and after drying, place them in a 6 cm dish containing 3-aminopropyltrimethoxysilane, and then place the slides in a 6 cm dish containing 0.5% NaOH. In a 6 cm dish of glutaraldehyde, prepare the solution according to previous study^25^. Place the cell slide on the bottom, gently drop an appropriate amount of polyacrylamide hydrogel solution onto the cell slide, and quickly cover the top of the droplet with another slide, solidify for 60 min. The polyacrylamide film surface was rinsed several times with 10 mM Hepes. Use 4 mL of 50 mM Hepes solution to dissolve 2 mg of Sulfo-SANPAH, and irradiate with UV light for 10 min. Take 0.2 mg/mL type I rat tail collagen, pipette an appropriate amount and evenly drop it on the surface of the treated polyacrylamide gel, and incubate at 4 °C overnight^25, 26^.

### Would healing and trans-well test

For would healing test, 5 × 10^5^ rat urethral primary fibroblasts were added to each culture dish, pictures were taken at 0, 6 h, 12 h and 24 h. For trans-well test, rat urethral primary fibroblasts were digested with trypsin, and the cell concentration was 3 × 10^5^ cells/mL in culture medium containing 1% fetal bovine serum (FBS). The upper chamber of the trans-well chamber was inoculated with 100 µL of cell suspension, and then 600 µL of culture medium containing 10% FBS was added to the lower chamber. And crystal violet staining was performed after culturing the cells for 24 h.

### Quantitative real-time polymerase chain reaction (qRT- PCR)

RNAiso Plus reagent (Takara Bio Inc, Otsu, Shiga, Japan) was used to extract total RNA and PrimeScript™ RT reagent kit (Takara Bio Inc Otsu, Shiga, Japan) was used to synthesized cDNA. SYBR Green Reagent (Takara, Kyoto, Japan) was used to perform the two-step real-time RT-PCR. The results were calculated by the 2^−ΔΔCt^ method and data were expressed as a ratio of the control gene GAPDH. Information on PCR primers is in Table S1.

### Western blotting

After trypsin digestion to extract the protein, the protein concentration was determined by Bicinchoninic Acid Assay (BCA) kit (Beyotime, Shanghai, China), electrophoresed on 10% color gel, transferred with PVDF membrane, and then blocked with 5% BSA (BioFroxx. Germany). Incubate the primary antibody at 4 °C overnight, incubate the corresponding secondary antibody, and develop ECL imaging. The antibodies used were as follows: Igfbp3 (Invitrogen) 1:1000; α-SMA (abcam) 1:1000; smad2 (CST) 1:1000; p-smad2 (CST); smad3 (CST) 1:1000; p-smad3 (CST); GAPDH (Abcam) 1:5000; α-tubulin (CST) 1:1000.

### mRNA sequencing

Total RNA was isolated and purified using TRIzol reagent (Takara, Kyoto, Japan). NanoDrop ND-1000 (NanoDrop, Wilmington, DE, USA) and Bioanalyzer 2100 (Agilent, CA, USA) was used for quality control. After fragment processing, the 2 × 150 bp paired-end sequencing (PE150) on an illumina Novaseq 6000 (LC-Bio Technology CO., Ltd., Hangzhou, China) was performed following the vendor's recommended protocol. The differentially expressed mRNAs were selected with fold change > 2 or fold change < 0.5 and with parametric F-test comparing nested linear models (*p* value < 0.05).

### Overexpression of lentivirus and siRNA

Igfbp3 (NM_012588-HA), the GV707 vector, the BamHI/XhoI cloning site, and the control CON522 were used for plasmid conduction. After digestion, the Igfbp3 fragment was amplified by PCR, transformed with competent E. coli, sequenced, and the plasmid was extracted (Table S2). The GV vector plasmid, pHelper 1.0 vector plasmid and pHelper 2.0 vector plasmid were mixed with 293T in a ratio of 4:3:2. siRNAs were designed and synthesized by manufacture (Shanghai Sangon Biotechnology, China) (Table S3).

### Cell transfected

Virus-infected cells were transfected into primary rat urethral fibroblasts with 100 MOI of virus solution, and siRNA was transfected with Lipofectamine 3000 (Invitrogen, Carlsbad, USA).

### Statistical analyses

All data were analyzed by SPSS 20.0 and Image J 1.8.0 software. According to the results of 3 or more independent repeated experiments, measurement data were expressed as mean ± standard deviation (mean ± SD), paired student’s T or one-way analysis of variance (ANOVA) was used for analysis, *p* < 0.05 was considered statistically significant, *p* < 0.05 (*); *p* < 0.01 (**) ; *p* < 0.001 (***); *p* < 0.0001 (****).

### Ethical approval

All procedures followed were in accordance with the ethical standards of the responsible committee on human experimentation (institutional and national) and with the Helsinki Declaration of 1975, as revised in 2000. Informed consent was obtained from all patients for being included in the study. All institutional and national guidelines for the care and use of laboratory animals were followed.

## Results

### Matrix stiffness in human urethral stricture increased

We enrolled three samples of urethral fibrosis from patients who had undergone surgery for urethral stricture, while normal urethral samples were collected from patients who underwent partial penile resection due to penile cancer, with their informed consent. Histological examination using H&E staining revealed distinct differences between the two groups. The urethral tissue in the normal group exhibited intact and well-organized epithelium, whereas the urethral stricture group displayed disordered tissue structure, with an unclear demarcation between the urothelial layer and the submucosa (Supplemental Fig. S1A). Moreover, Masson staining demonstrated disruption in the urethral structure of the stricture group, where collagen deposition was evident throughout the urethra, and connective tissue covered the urothelium near the lumen (Supplemental Fig. S1B).

**Figure 1:**
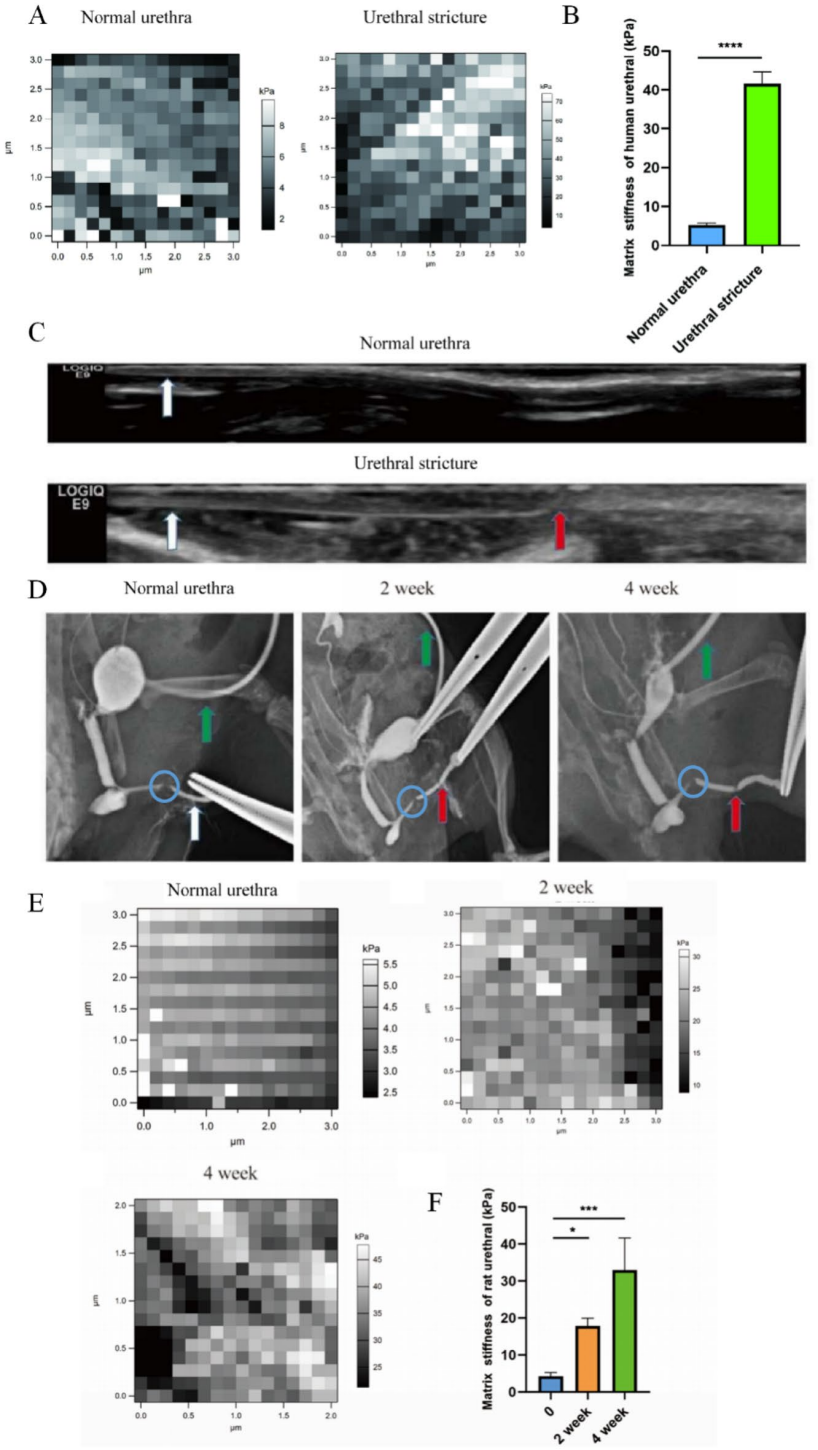
Matrix stiffness in urethral stricture rat models increased in a time-dependent manner. (**A**) AFM measurement of urethral tissue matrix stiffness; (**B**) Statistical analysis of matrix stiffness; (**C**) B-mode ultrasound of the rat urethra. The white arrows indicate the loach guide wire, and the red arrows indicate the urethral stricture; (**D**) Stricture modeling for 2 and 4 weeks, green arrows indicate fistulas. White arrows indicate normal anterior urethra, and red arrows indicate urethral strictures, the blue circle area is difficult for physiological contrast agents to pass through; (**F**) Statistical analysis of **E**. *p* < 0.05 (*); *p* < 0.001 (***); *p* < 0.0001 (****), n = 3. SPSS.

To quantitatively assess the stiffness of the urethra, we employed Atomic Force Microscopy (AFM) to analyze both normal and stricture urethra samples. The results demonstrated a notable contrast in matrix stiffness between the two groups. Specifically, the normal urethra exhibited a matrix stiffness of 5.23 ± 0.37 kPa, whereas the stricture urethra displayed significantly increased stiffness, reaching as high as 41.59 ± 2.48 kPa (Fig. 1A,B). These findings underscore the substantial difference in stiffness between normal and stricture urethral tissues, suggesting a potential link between matrix stiffness and the development of urethral fibrosis.

### Matrix stiffness in urethral stricture rat models increased in a time-dependent manner

The rat urethral stricture model was established by injuring rat urethral tissue. Compared with the normal urethra, the stricture urethra showed lower ultrasound echoes (Fig. 1C), and obstruction was felt at 1 cm from the urethral opening and epidural catheter could not pass through the urethra, indicating a urethral stricture. Similarly, compared with the normal urethra, the urethral stricture group (2 weeks and 4 weeks after urethral stricture modeling) had a contrast defect in the anterior urethra, indicating that the urethral stricture was formed here (Fig. 1D). It is worth noting that there is another contrast defect at the proximal end of the urethral stricture, which is the physiological curvature of the anterior urethra, due to the difficulty of the contrast agent staying (Fig. 1D, blue circle).

The rat urethral stricture model was further verified by H&E and Masson staining of rat urethral tissue. H&E staining showed that the urethral lumen and cavernosal vessels were narrowed in the urethral stricture group (Supplemental Fig. S2A). Masson staining showed that the rat urethral structure was disordered and collagen deposition in the urethral stricture group (Supplemental Fig. S2B). Two histological staining above further confirmed that the rat urethral stricture was successfully.

The rat urethral stricture samples collecting 2 and 4 weeks after modeling as well as normal urethra samples were used to detect the stiffness. The matrix stiffness of the normal rat urethra was 4.29 ± 0.82 kPa, while the matrix stiffness after urethral stricture modeling for 2 weeks was 17.87 ± 1.68 kPa, and the matrix stiffness after urethral stricture modeling for 4 weeks was 32.94 ± 7.12 kPa, indicating that matrix stiffness of rat urethral tissue increased a time-dependent manner (Fig. 1E,F). The results of rat models coincided with the clinical observation that fibrotic scars increasing with time in patients with urethral scars.

### Higher matrix stiffness stimulate FMT in urethral fibrosis

**Figure 2:**
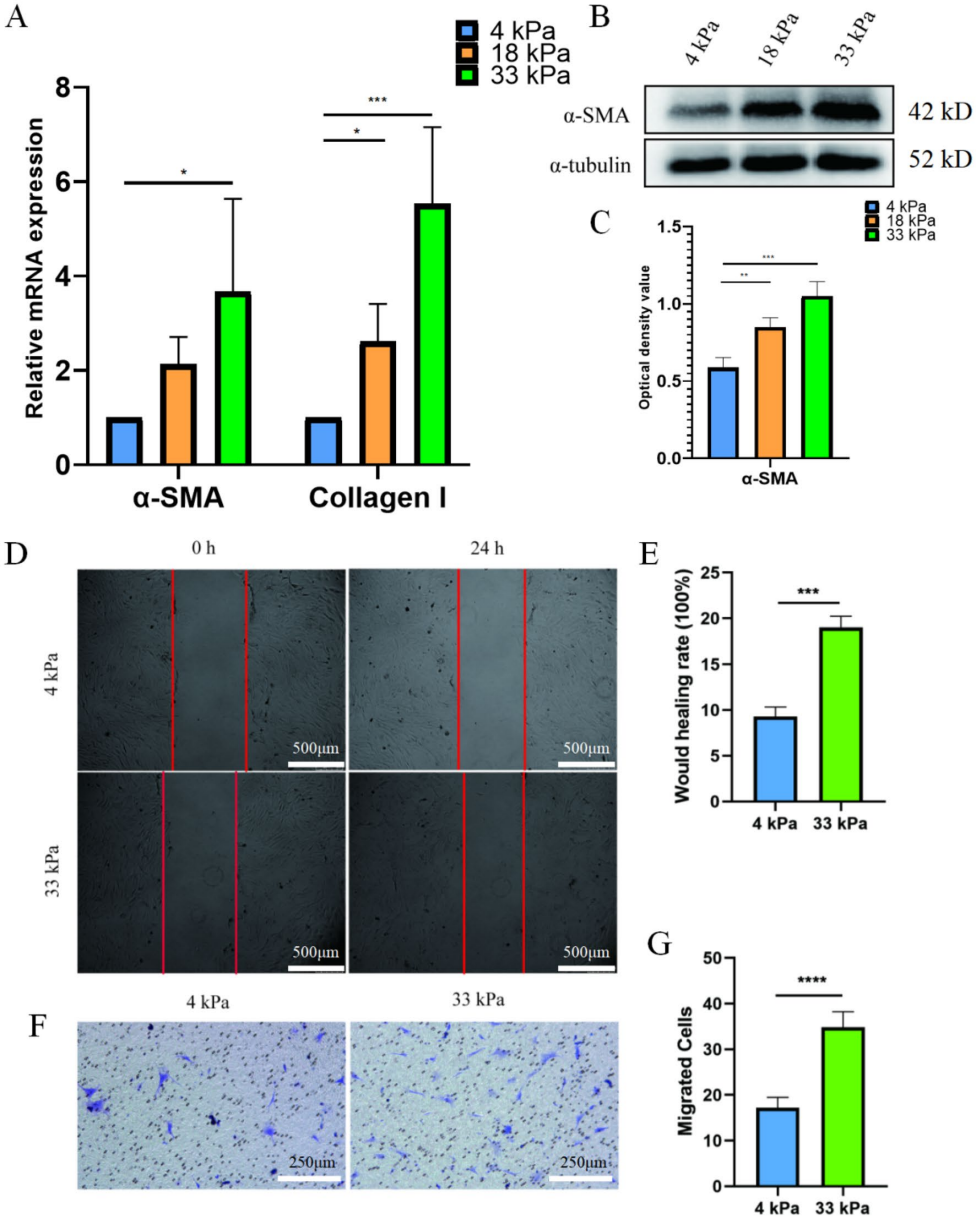
Higher Matrix stiffness stimulate FMT in urethral fibrosis. (**A**) qRT-PCR detection of α-SMA and Collagen I mRNA expression in primary rat urethral fibroblasts cultured with different extracellular matrix stiffness; (**B**) Western blotting; (**C**) Statistical analysis of **B**; (**D**) would healing test; (**E**) Statistical analysis of **D**, n = 3; (**F**) Transwell test; (**G**) statistical analysis of **E**, *p* < 0.001 (**); *p* < 0.001 (***); *p* < 0.0001 (****), n = 3. SPSS.

Primary rat urethral fibroblasts were isolated and identified (Supplemental Fig. S3). Diverse concentration of polyacrylamide hydrogel was used to mimic different matrix stiffness. Polyacrylamide hydrogels of 4 kPa, 16 kPa and 33 kPa were prepared to mimic the stiffness of urethra in the process of normal to 2 and 4 weeks after modeling, respectively. After culturing on hydrogels for 48 h, the expression of α-SMA and Collagen I increased in a stiffness-depend manner (Fig. 2A–C). After culturing on hydrogels for 48 h, fibroblasts were digested and passage to 6-well plates. The results showed that the healing index at an extracellular matrix stiffness of 33 kPa was significantly higher than that of primary rat urethral fibroblasts cultured at 4 kPa (Fig. 2D,E). Similar results were also observed in trans-well experiments, in which fibroblast culturing on 33 kPa hydrogel migrated more than those culturing on 4 kPa (Fig. 2F,G).

### Igfbp3 participated in urethral fibrosis

**Figure 3:**
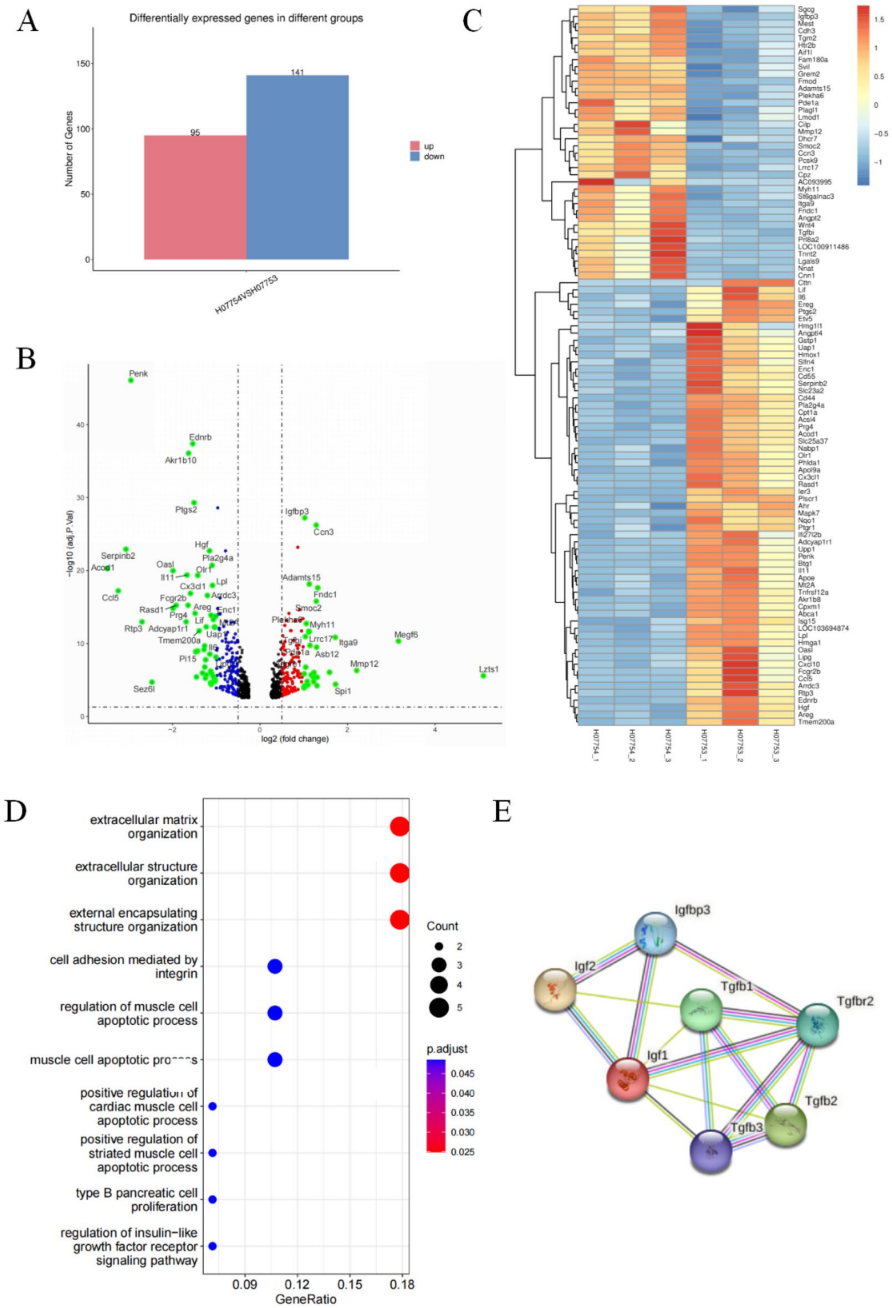
Transcriptomic analysis of different matrix stiffness. (**A**) Differentially expressed mRNA histogram; (**B**) differentially expressed mRNA volcano plot; (**C**) heat map of differentially expressed mRNA. Red indicates high mRNA expression, and blue indicates low mRNA expression; (**D**) KEGG analysis of upregulated genes; (**E**) predicted molecules related to Igfbp3 by STRING software. *p* < 0.05 (*); *p* < 0.01 (**); *p* < 0.001 (***), n = 3. SPSS.

mRNA was isolated from fibroblasts culturing on 4 kPa and 33 kPa hydrogels for 48 h, and transcriptomic analysis was performed. In the transcriptomic analysis of mRNA, a total of 95 mRNA expression levels were significantly increased and 141 mRNA levels were significantly decreased (Fig. 3A–C). We also performed GO and KEGG (www.kegg.jp/kegg/kegg1.html) analysis of differentially expressed mRNAs and qPCR validation of sequencing results (Figs. S4 and S5). In addition, GO analysis was constructed on the upregulated genes and found that they were related to multiple fibrosis pathways and extracellular matrix after clustering (Fig. 3D). PPI network using the upregulated genes was conducted. GO analysis results showed the positive regulation of insulin-like growth factor receptor signaling pathway (GO: 0043568) had the highest strength (Fig. S6A and C). The extracted genes including IGFBP3 IGFBP5 cdh3 RGD1309821. And previous studies have also suggested that the insulin-like growth factor receptor signaling pathway is closely related to fibrosis^27–31^. PPI analysis of 12 genes under GO: 0043568 showed that IGFBP3, IGFBP5, and CDH3 interacted with IGF1 (Fig. S6B). In addition, we conducted qRT-PCR on fibroblasts cultured at 4 kPa and 33 kPa, and found that IGFBP3, IGFBP5, CDH3, IGF1, and fibrosis indicators were increased (Fig. S6D).

Studies have reported that Igfbp3 could interact with TGF receptor, and TGF-β/Smad pathway is a classic pathway for fibrosis. Combined with the above analysis, it is speculated that Igfbp3 may play an important role in the increased fibrosis of rat urethral fibroblasts caused by increased matrix stiffness (Fig. 3E, STRING).

**Figure 4:**
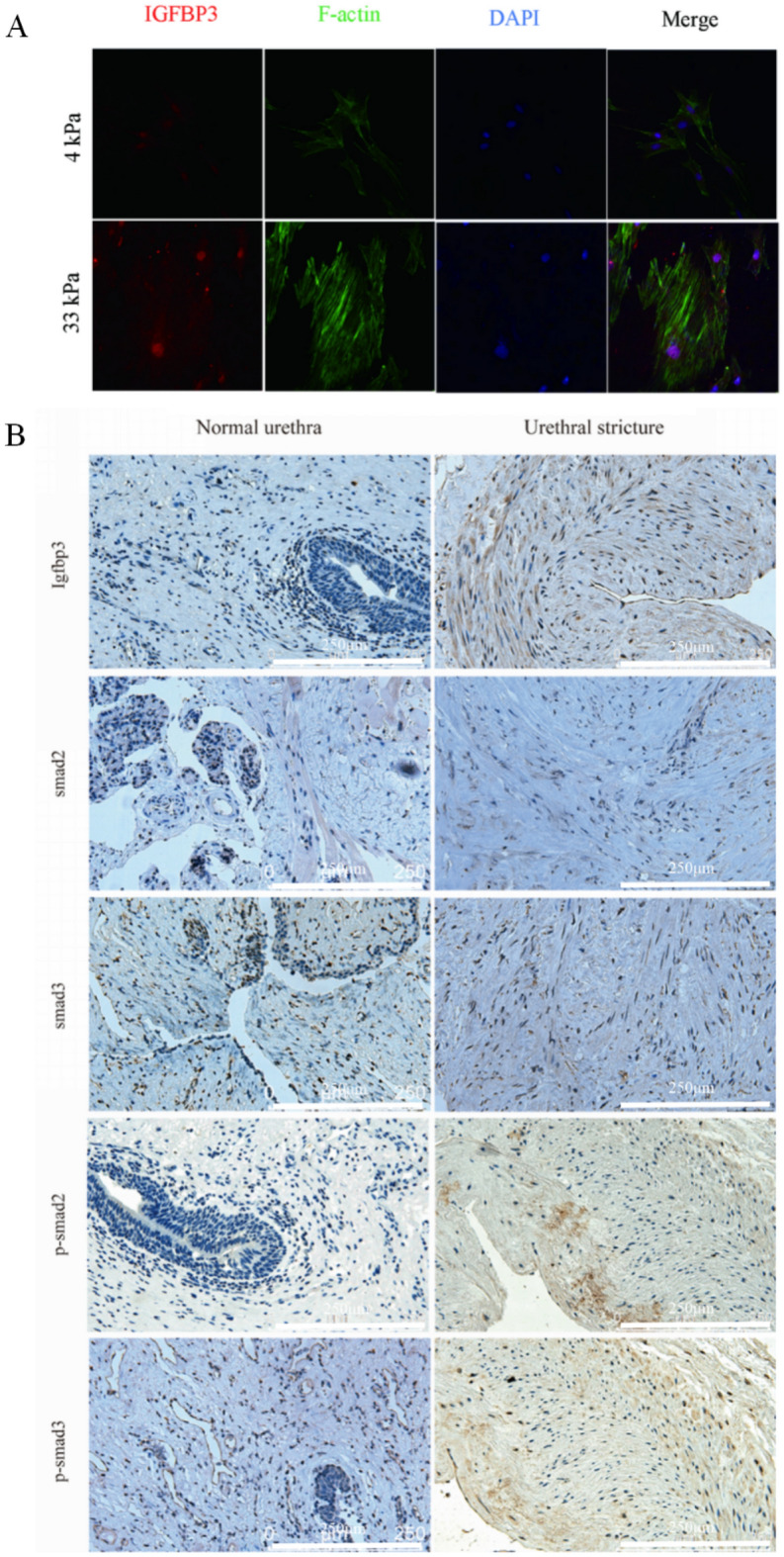
Igfbp3/Smad pathway participated in urethral fibrosis. (**A**) Immunofluorescence to determine the levels of Igfbp3 and F-actin at different matrix stiffness; (**B**) expression of Igfbp3/Smad pathway in human urethral stricture tissue and normal urethral tissue.

Immunofluorescence was performed to measure the expression of Igfbp3 after culture in different matrix stiffness, and measured the distribution of actin in cells. It was found that cells cultured in high matrix stiffness had higher expression of Igfbp3, larger cell area, lower roundness, and thicker actin (Fig. 4A). IHC was performed on human normal urethral tissue and urethral stricture tissue. In the urethral stricture tissue, the expression of Igfbp3 was higher than that in the normal urethral group, the expression of smad2 and smad3 seemed unchanged, while the expression levels of p-smad2 and p-smad3 in the urethral stricture tissue were higher than normal urethral tissue (Fig. 4B).

**Figure 5:**
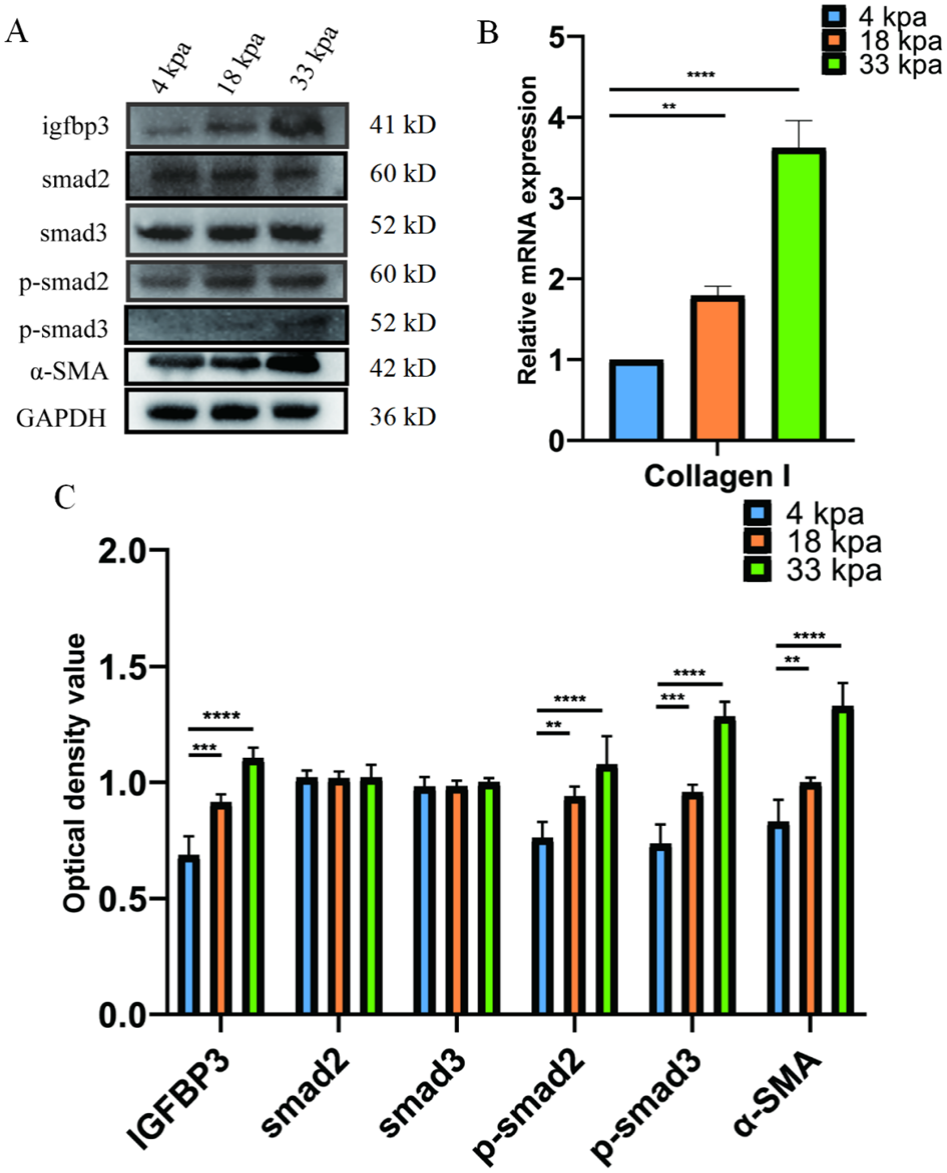
Extracellular matrix stiffness affects urethral stricture progression through regulation of Igfbp3/pathway. (**A**) Western blotting; (**B**) qRT-PCR; (**C**) Statistical analysis of **A**; *p* < 0.01 (**); *p* < 0.001 (***); *p* < 0.0001 (****); n = 3. SPSS.

Rat urethral primary fibroblasts were culture on the 4 kPa and 33 kPa for 48 h. qRT-PCR was used to verify the mRNA expression of Collagen I (Fig. 5B). Fibrosis markers and Igfbp3/Smad protein expression levels were detected by Western blotting (Fig. 5A, C). The results showed that with the increase of extracellular matrix stiffness, the expression of Igfbp3, p-smad2, p-smad3 and the fibrosis indicator α-SMA were increased. However, the levels of total smad2 and smad3 did not show a statistically significant change of extracellular matrix stiffness.

### Matrix stiffness accelerate FMT through Igfbp3/Smads

**Figure 6:**
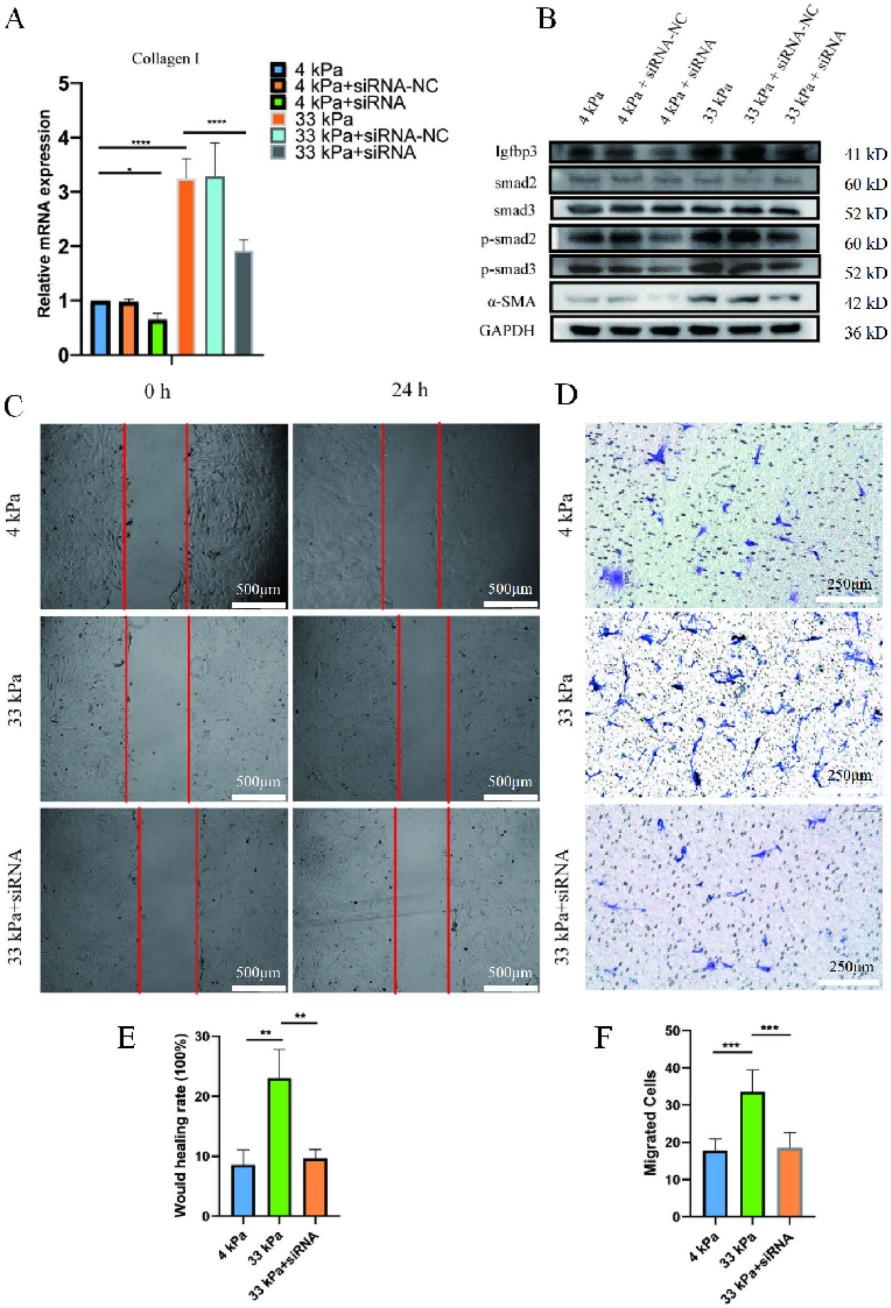
Igfbp3-siRNA inhibits the matrix stiffness-induced FMT. (**A**) qRT-PCR; (**B**) Western blotting; (**C**) Would healing test; (**D**) Transwell test; (**E**) Statistical analysis of **C**; (**F**) Statistical analysis of **D**. *p* < 0.05 (*); *p* < 0.01 (**); *p* < 0.001 (***); *p* < 0.0001 (****); n = 3. SPSS.

Previous studies indicated that Igfbp3 might interacted with TGF-β^32^, thus, we checked whether Igfbp3 might regulated the expression of Smads in fibroblast. When inhibited the expression of Igfbp3 in fibroblast, the expression of α-SMA and Collagen I decreased in both fibroblast culturing on 4 and 33 kPa gels (The inhibitory ability of siRNA is shown in Fig. S7D and E). Accelerating FMT in 33 kPa gels can be partially reversed by interfering the expression of Igfbp3. The expression of Smad2, Smad3, P-Smad2 and P-Smad3 were decreased in Igfbp3 interfering fibroblast (Fig. 6A,B). The wound healing test and trans-well experiment also demonstrated that inhibiting Igfbp3 could reduce the migration ability of fibroblasts cultured on high stiffness (Fig. 6C–F).

**Figure 7:**
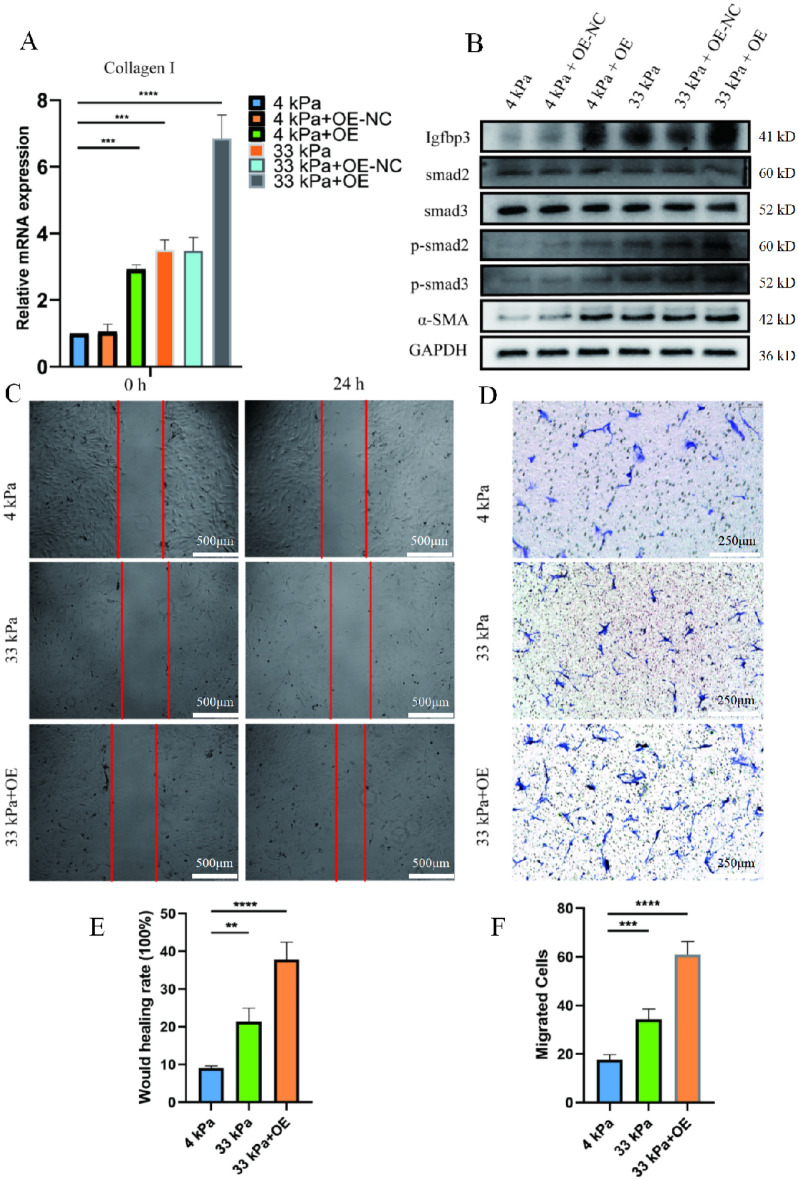
Igfbp3-overexpression prometes the matrix stiffness-induced FMT. (**A**) qRT-PCR; (**B**) Western blotting; (**C**) Would healing test; (**D**) Transwell test; (**E**) Statistical analysis of **C**; (**F**) Statistical analysis of **D**. *p* < 0.05 (*); *p* < 0.01 (**); *p* < 0.001 (***); *p* < 0.0001 (****); n = 3. SPSS.

To further prove that the extracellular matrix stiffness induce urethral fibroblast activation and aggravation of fibrosis through the Igfbp3/Smad pathway, we overexpression Igfbp3 in fibroblast (The overexpression ability of Lentivirus is shown in Fig. S7A–C). The expression of α-SMA and Collagen I increased in both fibroblast culturing on 4 and 33 kPa gels, and we also observed increased migration even in fibroblast culturing on 4 kPa. Although the expression of Smad2 and Smad3 remained unchanged, however, the expression of P-Smad2 and P-Smad3 increased (Fig. 7A,B). In wound healing test and trans-well experiment also demonstrated that overexpressed Igfbp3 could enhance the migration ability of fibroblasts cultured on high stiffness (Fig. 7C–F).

## Discussion

In this study, we utilized AFM to measure the stiffness of human urethral stricture scars. Previously, the stiffness of lung fibrosis, renal fibrosis tissue have been measured with AFM, while there has been a dearth of reports regarding the stiffness of human urethral tissue^12, 13^. In the non-invasive measurement by Contrast-Enhanced Ultrasound and Shear Wave Elastography the matrix stiffness of the corpus cavernosum was 32.6 ± 5.4 kPa in the stenosis of the bulbar urethra, and 27.3 ± 5.8 kPa in the corpus cavernosum near the stenotic tissue. However, these findings do not provide specific information about the stiffness of the urethra or the urethral stricture scar itself^33^. Therefore, our study stands as a pioneering effort in reporting the stiffness of both normal human urethra and fibrotic urethra.

A rat urethral stricture model had been constructed in this study to mimic the pathogenesis of urethral fibrosis. By AFM, we measured the stiffness of normal and fibrotic urethral tissues in both rat and human samples. The results revealed that the stiffness of the normal rat and human urethra was almost identical, measuring at 4.29 ± 0.82 kPa and 5.23 ± 0.37 kPa, respectively. However, 4 weeks after surgery, the stiffness of rat model urethra reached to 32.94 ± 7.12 kPa, approximating the stiffness observed in human stricture tissues (41.59 ± 2.48 kPa). These findings provide valuable insights into the stiffening process during the progression of urethral fibrosis in the rat model and offer a significant comparison to human stricture tissues.

Tissue mechanics, encompassing aspects such as matrix stiffness, tension, hydrostatic forces, tensile forces, and osmotic pressure play a crucial role in fibrosis. These forces together regulate various cellular processes, including the proliferation of fibroblasts and other cell types, the activation of growth factors in damaged tissue as well as the structure and forces of the matrix^34^. Among them, the investigation of matrix stiffness in relation to fibrosis has garnered the most extensive and in-depth attention. The definition of matrix stiffness in biology and biomedicine is the ability of an object to resist deformation by external forces^34^. Extensive research has explored the interplay between matrix stiffness and fibrosis in various tissue and organ settings, such as pancreatic fibrosis, myocardial fibrosis, liver fibrosis, and wound healing. In these studies, higher matrix stiffness was consistently associated with enhanced fibroblast proliferation, increased migratory capabilities, and elevated extracellular matrix (ECM) expression^35–37^. Our study yielded similar findings, confirming the influence of matrix stiffness on fibrosis progression. Moreover, by using cellular and animal models, we observed a time-dependent advancement of fibrosis, closely correlated with the increasing stiffness of the micro-environment. The evidence from our study, in conjunction with existing literature, further substantiates the significance of matrix stiffness as a key modulator of fibrosis. Understanding the intricate relationship between tissue mechanics and fibrotic processes holds promising implications for devising targeted therapeutic approaches aimed at mitigating fibrosis progression in various pathological conditions.

Studies have shown that matrix stiffness plays a critical facilitating role in regulating TGFβ1-stimulated lung fibroblast contraction, suggesting that stiff fibrotic lung tissue may promote myofibroblast activation through contraction, whereas normal lung tissue compliance could prevent formation of lung myofibroblasts^38^. In the present research, there are four main pathways for the molecular mechanism of ECM stiffness to enhance the degree of fibroblast fibrosis and to activate fibroblasts. (1) Integrin-related pathways^17, 18^; (2) focal adhesion kinase (FAK) related pathway^19^; (3) RhoA/Rho-ROCK related signaling pathway^20^; (4) YAP/TAZ related signaling pathway^21^. In addition, mechanosensitive ion channels such as PIEZO1, transient receptor potential ion channels vanilloid receptor 4 (TRPV4) and the lysyl oxidase (LOX) family have also been shown to play an important role in matrix stiffness promoting fibrosis^22, 23^. Although numerous studies have shown that extracellular matrix stiffness can activate fibroblasts, including fibroblasts in multiple organs such as lung, heart, kidney, and skin, the specific impact and underlying mechanism of matrix stiffness on urethral fibroblasts remain an area necessitating further investigation.

Through bioinformatics analysis, Igfbp3 might contributed to regulating FMT and promoting the progression of urethral fibrosis. FMT contributed to the pathogenesis of fibrosis. Compared with fibroblast, myofibroblast secreted more ECM component and α-SMA. Extra ECM increased the stiffness of matrix and higher expression of α-SMA increased the contraction of scar, which stimulate FMT in turn. We also observed a stiffness-depend advanced progression of FMT in our study.

Bioinformatic analysis has implicated Igfbp3 as a potential regulator of fibrosis through its involvement in TGF-β signaling pathways^39^. Igfbp3 is found to be high expression in urethra and bladder^40^. Igfbp3 can combine with insulin-like growth factor and play a role in synergistic regulation of cell proliferation and apoptosis. Igfbp3 is closely related to fibrosis. Studies on patients with idiopathic pulmonary fibrosis have revealed a significant increase in Igfbp3 content in bronchoalveolar lavage fluid, lung tissue, and lung primary fibroblasts, where it exerts a promoting effect on extracellular matrix deposition^41^. Similarly, our investigation unveiled elevated Igfbp3 levels in human urethral stricture tissue.

In cellular and animal models, we evaluated the expression of Smads proteins, key components of the classical TGF-β signaling pathway. Notably, when we inhibited Igfbp3 expression, p-Smad2/3 levels decreased, while overexpression of Igfbp3 in fibroblasts led to increased p-Smad2/3 levels. This finding led us to believe that Igfbp3 is involved in fibrosis through the regulation of Smad2/3.

However, the precise mechanism through which Igfbp3 senses mechanical stimulation remains incompletely elucidated in this study. It is conceivable that Igfbp3 might interact with proteins that are sensitive to matrix stiffness, such as FAK and LOX, both of which serve as potentially crucial regulators of gene expression in cancer, influencing cell function and shaping the surrounding tumor microenvironment. Addressing this aspect is an avenue that will receive focused attention in our future studies. By further investigating the interplay between Igfbp3 and these mechanosensitive proteins, we aim to gain a more comprehensive understanding of how matrix stiffness influences fibrosis and related cellular responses.

## Conclusion

In sum, Igfbp3/Smad pathway participates in driving the progressive exacerbation of urethral fibrosis and Igfbp3 might be a novel therapeutic target for urethral stricture.

### Supplementary Information

Supplementary Information.

## Data Availability

All relevant data are included in the manuscript. Materials, data, and protocols described within the paper are available upon reasonable request to the corresponding authors.
