# Supplementary material for: Extracellular matrix stiffness aggravates urethral stricture through Igfbp3/Smad pathway
Source: Sci Rep. 2023 Aug 31;13:14315. doi: 10.1038/s41598-023-41584-6 (PMC10471624; doi:10.1038/s41598-023-41584-6)
Supplement: Supplementary file 1 — Supplementary Information. [file 41598_2023_41584_MOESM1_ESM.docx]

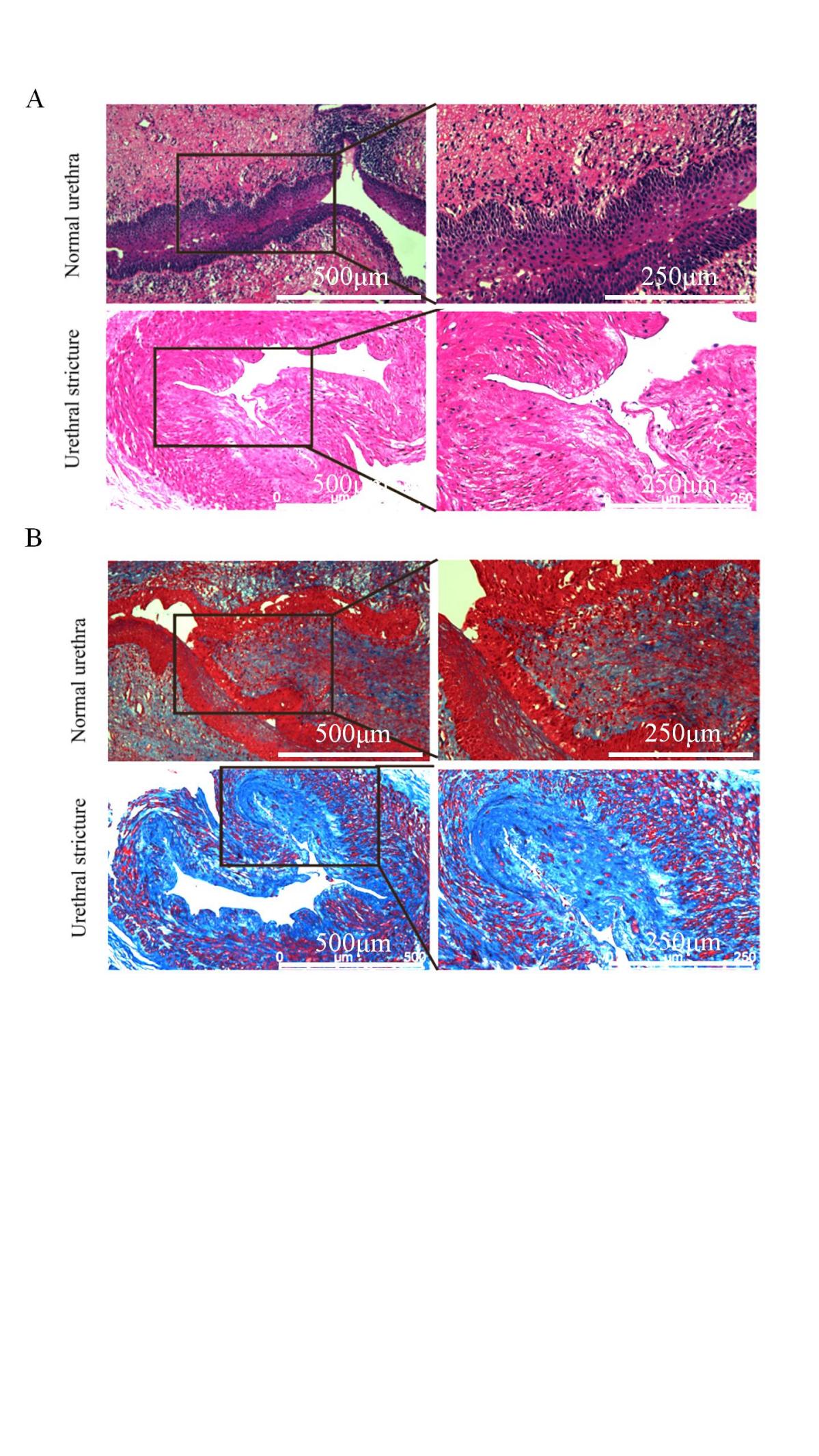


Figure S1. Histological staining of human normal urethra and stricture urethra.

(A) H&E staining and (B) Masson staining of normal and urethral stricture tissues.


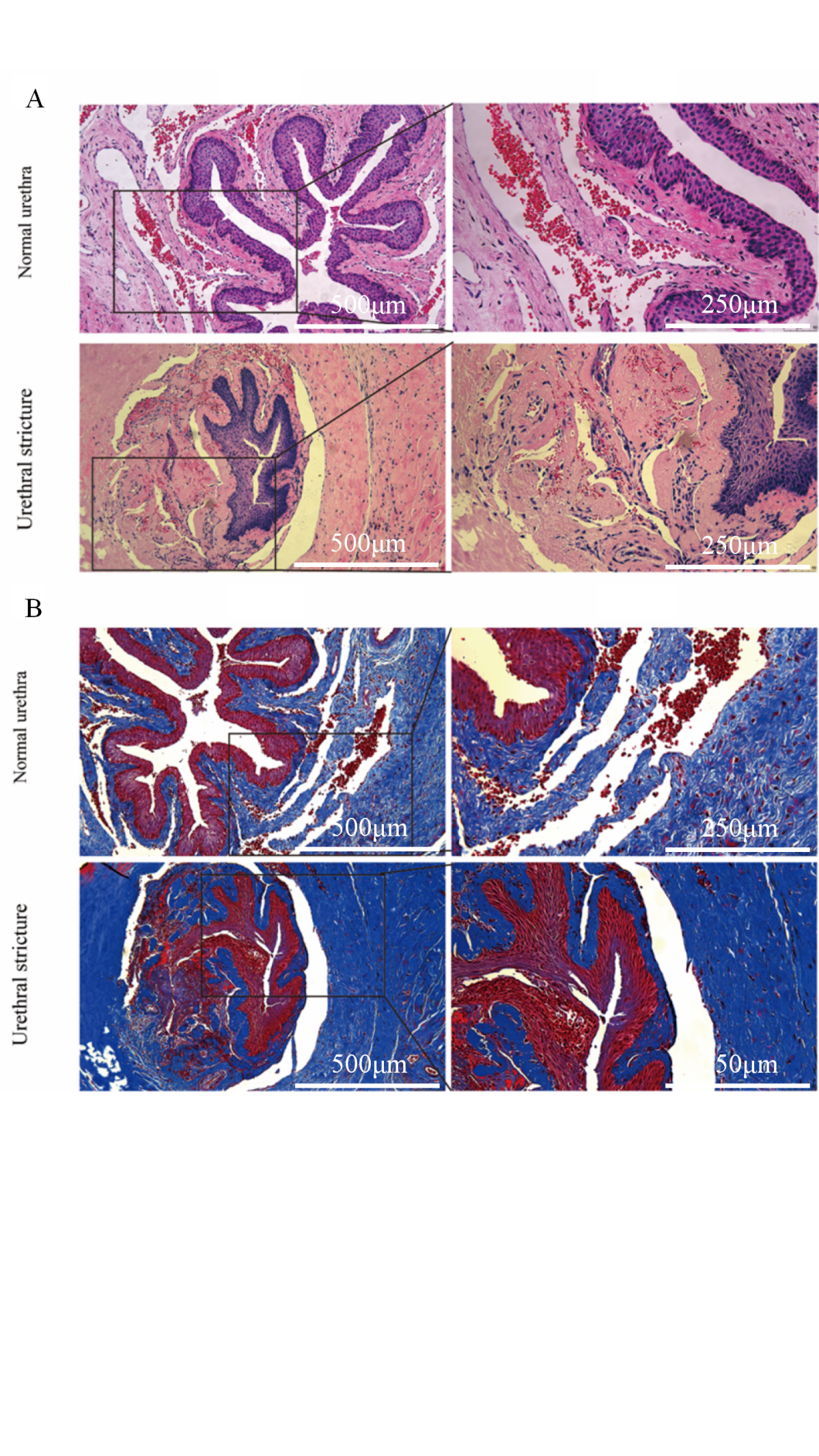


Figure S2. Validation of the urethral stricture model.

(A) H&E staining of normal rat urethra and stricture urethra; (B) Masson staining of rat normal and stricture urethra.


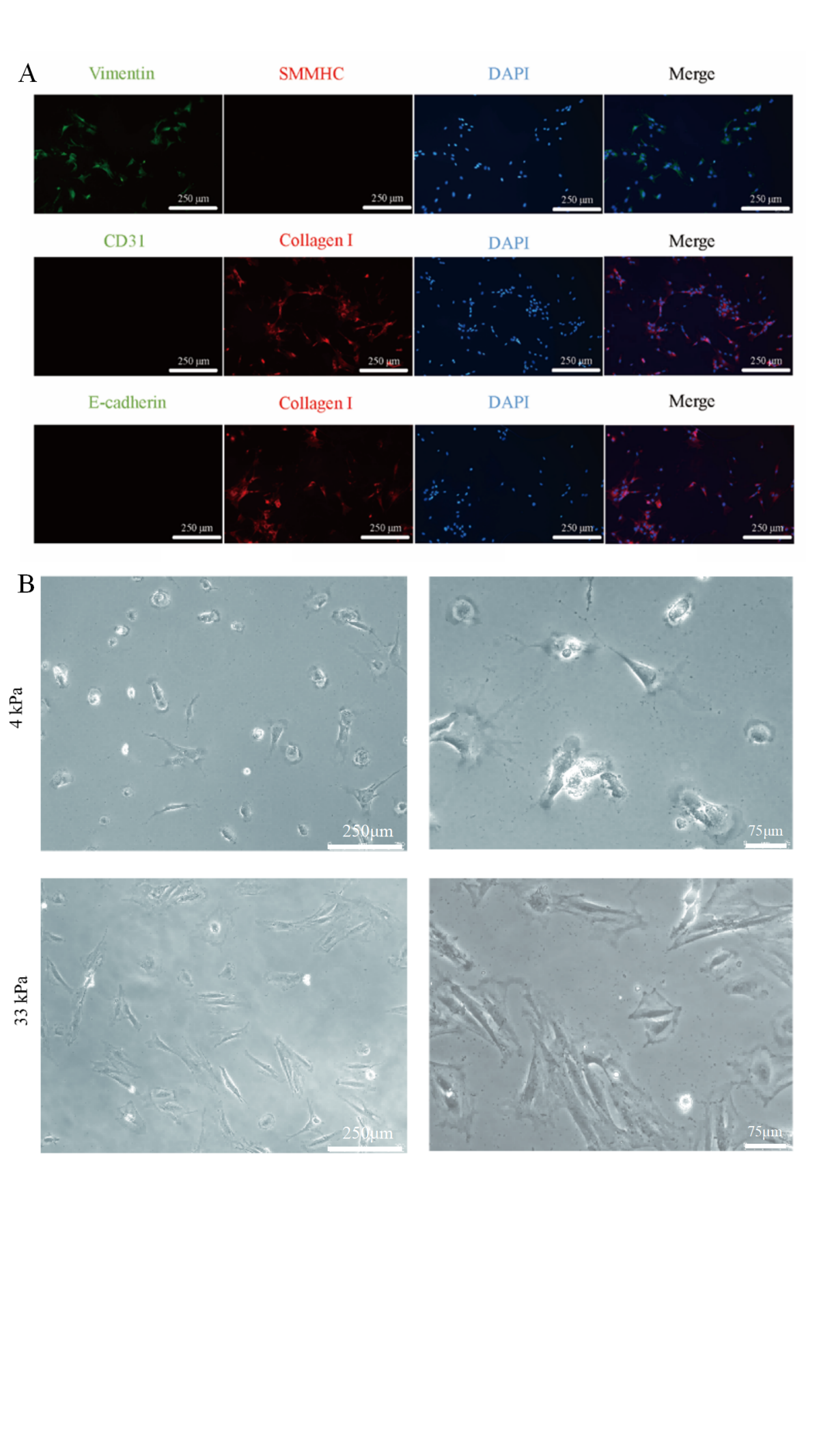


Figure S3. Fibroblasts extracted from rat urethra.

1. Rat urethral primary fibroblasts were identified by immunofluorescence, and the measured indexes were Vimentin/SMMHC, CD31/Collagen I, E-cadherin/Collagen I. (B) The morphology of fibroblasts cultured under different matrix stiffness.


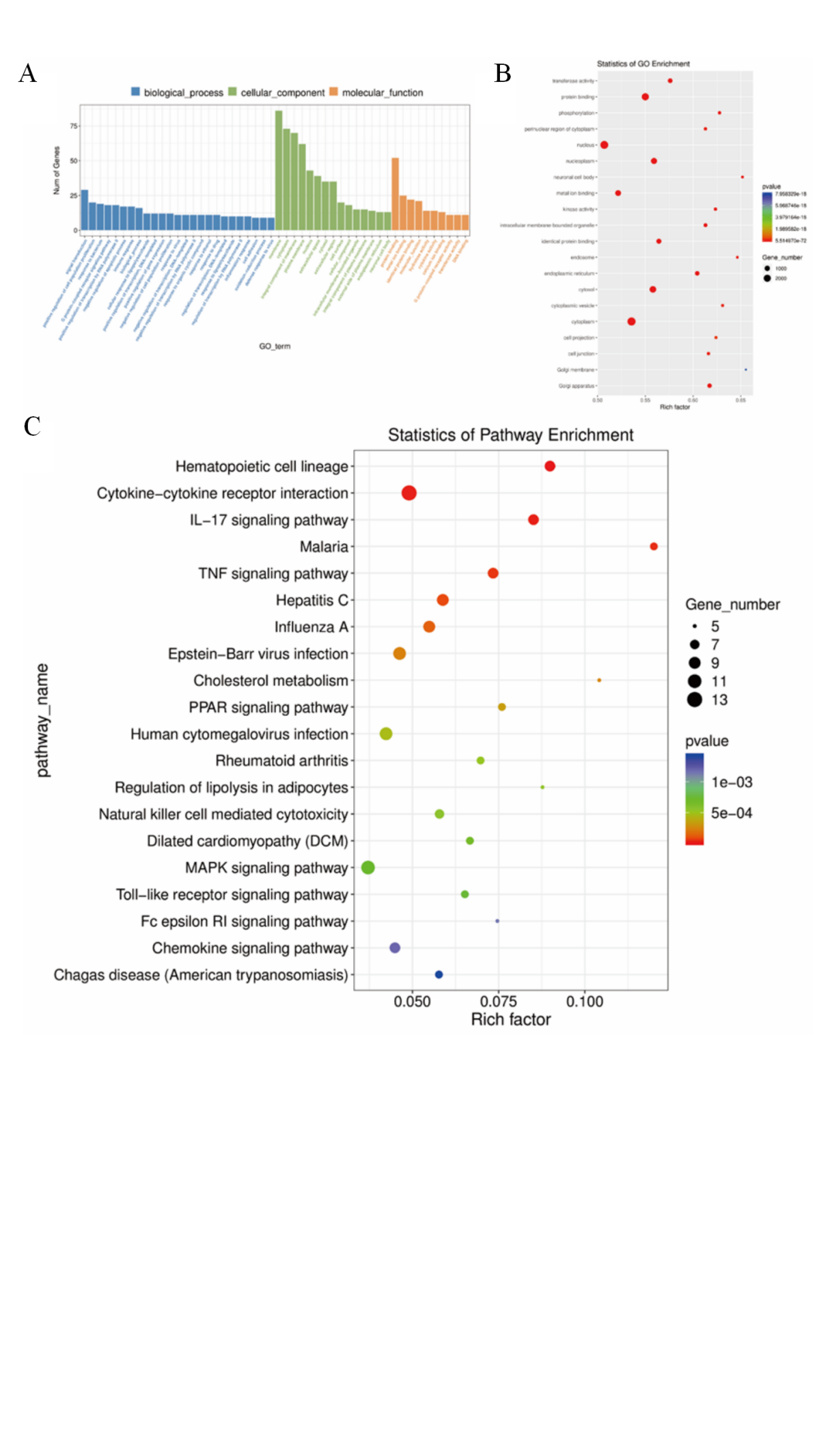


Figure S4. GO and KEGG enrichment analysis of differential genes.

(A) Enrichment of differential genes on molecular functions, biological processes and cell composition; (B) Top20 GO differential genes; (C) Top20 KEGG enrichment analysis.


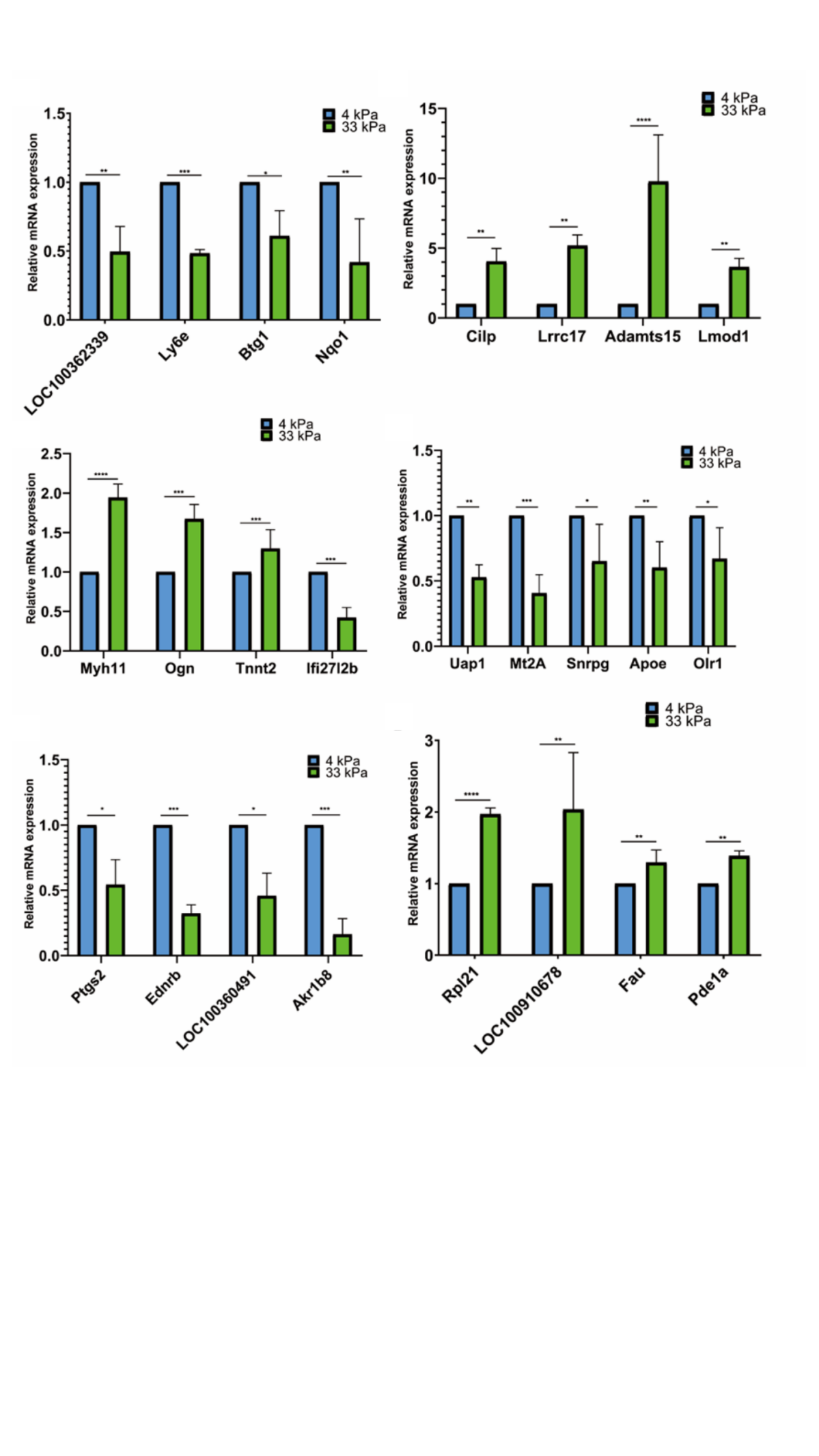


Figure S5. Verification of transcriptomic sequencing results.

qRT-PCR validation of up- and down-regulated mRNAs in sequencing results; p<0.05 (*); p<0.01 (**); p<0.001 (***); p<0.0001 (****); n=3.


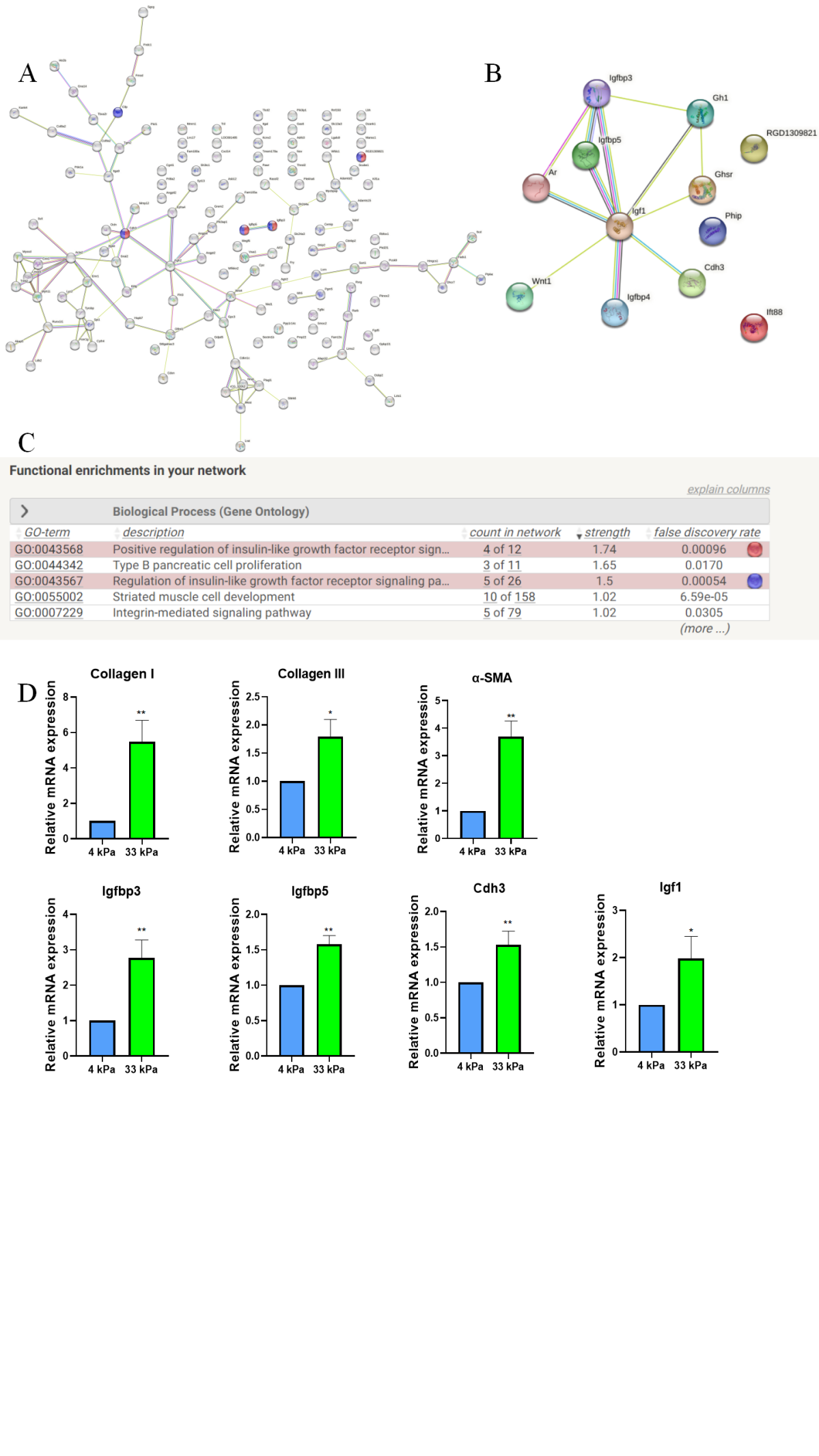


Figure S6. Transcriptomic analysis of different matrix stiffness.

(A) PPI network for upregulating genes; (B) PPI analysis of 12 genes under GO: 0043568; (C) GO analysis of A; (D) q-RT PCR validation;p<0.05 (*); p<0.01 (**); n=3.


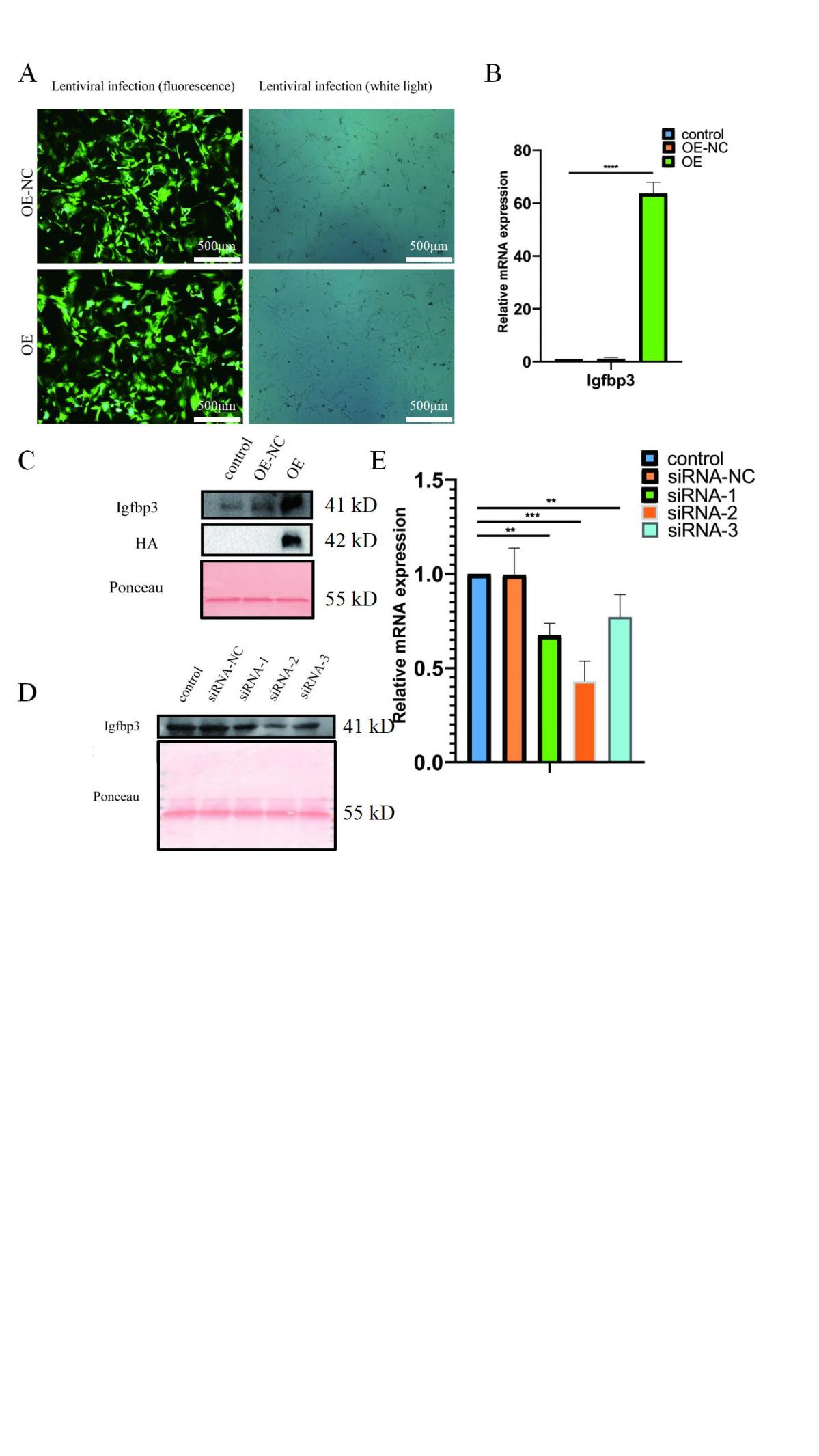


Figure S7. Validation of the ability of Igfbp3 siRNA and overexpression in rat urethral primary fibroblasts.

(A) Rat urethral primary fibroblasts were transfected with lentivirus overexpressing Igfbp3, MOI=100, left picture: green fluorescence, right picture: white light; (B) qRT-PCR detected the mRNA expression of Igfbp3 after overexpression; (C) The expression of Igfbp3 and HA detected by Western blotting; (D) Western blotting detection of Igfbp3 protein expression in primary rat urethral fibroblasts after interference with Igfbp3; (E) qRT-PCR detection of Igfbp3 mRNA expression in rat primary urethral fibroblasts after interference with Igfbp3; p<0.01 (**); p<0.001 (***); p<0.0001 (****); n=3.

Table S1. qRT-PCR Primers

| Primers | Sequencing |
| --- | --- |
| Rpl21-Rat-F | GTAGACATCAAGGGAATGGGCACTG |
| Rpl21-Rat-R | GCTGGGTGACATTGTAGACTCTTCC |
| LOC100910678-Rat-F | AGGAGATGGACGAGGAAGATAAGGC |
| LOC100910678-Rat-R | CGGCCTTGGCTTTTAGCTCCTC |
| Fau-Rat-F | AAGTGAGAGGTCAGACTCCCAAGG |
| Fau-Rat-R | AGCGTCGGTTGTACTGCATTCG |
| Pde1a-Rat-F | TTCCATGTTGCTGACGCTCTGAG |
| Pde1a-Rat-R | CCACCTCTCCTTGTTCTGCTGAATG |
| Myh11-Rat-F | GATCAAGGAGCGGCAGCAGAAG |
| Myh11-Rat-R | GCATCTCTTCAGCCTCAGCATACAG |
| Ogn-Rat-F | GATGAAATGCCCACATGCCTGTTG |
| Ogn-Rat-R | TTTGGCAGTGGTGGTACAGCATC |
| Tnnt2-Rat-F | CGTGAGGAGGAGGAGAACAGGAG |
| Tnnt2-Rat-R | TCTCTTCCCACTCTTCCGCTCTG |
| Cilp-Rat-F | GAGTTCCTGGCTTGTGTCCTGATG |
| Cilp-Rat-R | CTCTGCGTTGATGGTGGCTGAC |
| Lrrc17-Rat-F | ATGAAGAGGAGAAGGAGCGACTGG |
| Lrrc17-Rat-R | CGTAATTGTGGCACAGAGTGGAGTC |
| Adamts15-Rat-F | AAATGTGGCGTGTGTGGTGGAG |
| Adamts15-Rat-R | CTGGCGGATGTCAATGCTGGAG |
| Lmod1-Rat-F | TGGAGGCAAGACGGAGATGGAG |
| Lmod1-Rat-R | TTCGGCTAAGCAGATTGGTGACAG |
| LOC100362339-Rat-F | ACCAGCAGGAGTTCGTCAGAGC |
| LOC100362339-Rat-R | AACTTGACTGTGTCCACCCATTCG |
| Ly6e-Rat-F | GTCCATTCCCTGATGTGCTTCTCC |
| Ly6e-Rat-R | GGTGTAGCCAAGGTTGACATTCCC |
| Btg1-Rat-F | GCGGTGTCCTTCATCTCCAAGTTC |
| Btg1-Rat-R | GCAGGCTCTGGCTGAAAGTCTG |
| Nqo1-Rat-F | AGGATGGGAGGTGGTCGAATCTG |
| Nqo1-Rat-R | GCCTTCCTTATACGCCAGAGATGAC |
| Ifi27l2b -Rat-F | GGCTTCACTGGGTCAGGCATTG |
| Ifi27l2b -Rat-R | TGTAGTGTGGCTACCAGGCTTCC |
| Uap1-Rat-F | AGGATGCCAATGATGTGCCGATC |
| Uap1-Rat-R | GCCACATAGCCTTCAAGTCCTTCTC |
| Mt2A-Rat-F | TGGCTCCTGCAAATGCAAACAATG |
| Mt2A-Rat-R | TGCACTTGTCCGAAGCCTCTTTG |
| Snrpg-Rat-F | AACCTCGTGATTGATGAGTGTGTGG |
| Snrpg-Rat-R | TGATGATGCTGTTTCCTCGGATGAC |
| Apoe-Rat-F | AGGAGCAGACCCAGCAGATACG |
| Apoe-Rat-R | GCAATGGAGTTGGTAGCCACAGAG |
| Olr1-Rat-F | AGATAGACACCCTCACCTGGAAGC |
| Olr1-Rat-R | CACTTGGACTCCTCTGAAGCGTTC |
| Ptgs2-Rat-F | CACATTTGATTGACAGCCCACCAAC |
| Ptgs2-Rat-R | AGTCATCAGCCACAGGAGGAAGG |
| Ednrb-Rat-F | AGTCGTGTTTGTGCTGCTGGTG |
| Ednrb-Rat-R | GCTGGAGCGGAAGTTGTCGTATC |
| LOC100360491-Rat-F | GCCATCACGGAAGAGGAGAAGAAC |
| LOC100360491-Rat-R | TCTTGCTCTGCGGCTTCTTTCG |
| Akr1b8-Rat-F | TCGTGGTCACAGCCTACAGTCC |
| Akr1b8-Rat-R | TCTTGTGCTTGGCGGCAATCTC |
| Igfbp3-Rat-F | AGAAACAGTGTCGCCCTTCCAAAG |
| Igfbp3-Rat-R | AGGCAATGCACGTCGTCTTTCC |
| Col-1-Rat-F | TGATCGTGGTGATACTGTCCTG |
| Col-1-Rat-R | CTTTATGCCTCTGTCGCCCTGTTC |
| α-SMA-Rat-F | CGTGGCTATTCCTTCTGTACTACTG |
| α-SMA -Rat-R | CGTCAGGCAGTTCGTAGCTCTTC |
| Gapdh-Rat-F | TGGAGTCTACTGGCGTCTT |
| Gapdh-Rat-R | TGTCATATTTCTCGTGGTTCA |

Table S2. Positive clone of Igbfp3 sequences

| Positive clone of Igbfp3 |
| --- |
| TCCCAGGTCCAACTGCACCTCGGTTCTAAGCTTCTGCAGGTCGACTCTAGAGGATCCCGCCACCATGCATCCCGCGCGCCCCGCGCTCTGGGCGGCTGCGCTCACCGCCCTCACTCTGCTCCGCGGACCGCCAGTGGCGCGGGCCGGCGCGGGCGCGGTGGGCGCGGGCCCCGTGGTGCGCTGCGAACCGTGCGACGCGCGTGCGCTGGCCCAGTGCGCGCCTCCGCCCACCGCGCCCGCGTGCACGGAGCTGGTGCGAGAACCCGGCTGCGGCTGCTGCCTGACTTGCGCGCTGCGCGAAGGCGACGCGTGCGGCGTCTACACGGAGCGCTGTGGCACCGGCCTCCGCTGCCAGCCGCGACCGGCCGAGCAGTATCCCCTGAAGGCGCTGCTGAATGGCCGCGGGTTCTGCGCCAACGCCAGCGCCGCCAGCAGCCTGAGTGCCTACCTCCCCTCCCAGCCGTCTCCTGGAAACACCACTGAGTCTGAGGAGGACCACAATGCTGGGAGTGTGGAAAGCCAGGTTGTCCCCAGCACACACCGCGTGACTGATTCCAAGTTCCATCCACTCCATTCAAAGATGGAGGTCATCATAAAAGGCCAGGCTAGGGACAGCCAGCGCTACAAAGTTGACTATGAGTCCCAGAGCACAGACACCCAGAACTTCTCCTCCGAGTCTAAGCGGGAGACAGAATATGGTCCCTGCCGCAGAGAAATGGAGGACACACTGAATCATCTGAAGTTCCTCAATGTGCTGAGTCCCAGGGGCGTCCACATCCCAAACTGTGACAAGAAGGGGTTCTATAAGAAGAAACAGTGTCGCCCTTCCAAAGGCAGAAAGCGGGGCTTCTGCTGGTGCGTGGACAAGTACGGGCAGCCATTGCCAGGCTATGACACCAAGGGGAAAGACGACGTGCATTGCCTCAGCGTGCAGAGCCAGGGATCATACCCTTATGATGTCCCAGACTATGCTTAGCTCGAGTCCATCGATACTAGTAAGGATCTGCGATCGCTCCGGTGCCCGTCAGTGGGCAGAGCGCACATCGCCCACAGTCCCCGAGAAGTTGGGGGGAGGGGTCGGCAA |

Table S3. siRNA sequences

| siRNA | sequences |
| --- | --- |
| siRNA-1-RIgfbp3-658-forward | GCGCUACAAAGUUGACUAUTT |
| siRNA-1-RIgfbp3-658-reverse | AUAGUCAACUUUGUAGCGCTT |
| siRNA-2-RIgfbp3-757-forward | GGAGGACACACUGAAUCAUTT |
| siRNA-2-RIgfbp3-757-reverse | AUGAUUCAGUGUGUCCUCCTT |
| siRNA-3-RIgfbp3-861-forward | GCCCUUCCAAAGGCAGAAATT |
| siRNA-3-RIgfbp3-861-reverse | UUUCUGCCUUUGGAAGGGCTT |
| siRNA-NC-forward | UUCUCCGAACGUGUCACGUTT |
| siRNA-NC-reverse | ACGUGACACGUUCGGAGAATT |
